# 淋巴结清扫方式对非小细胞肺癌患者术后慢性咳嗽的影响

**DOI:** 10.3779/j.issn.1009-3419.2025.102.24

**Published:** 2025-06-30

**Authors:** Zekai ZHANG, Gaoxiang WANG, Zhengwei CHEN, Mingsheng WU, Xiao CHEN, Tian LI, Xiaohui SUN, Mingran XIE

**Affiliations:** 230001 合肥，中国科学技术大学附属第一医院胸外科; Department of Thoracic Surgery, The First Affiliated Hospital of University of Science and Technology of China, Hefei 230001, China

**Keywords:** 肺肿瘤, 淋巴结清扫方式, 慢性咳嗽, 中文版莱斯特咳嗽问卷, Lung neoplasms, Lymph node dissection method, Chronic cough, Mandarin Chinese version of the Leicester Cough Questionnaire

## Abstract

**背景与目的** 肺癌是所有恶性肿瘤中致死率排名最高的恶性肿瘤，其中非小细胞肺癌（non-small cell lung cancer, NSCLC）占所有肺癌的80%-85%。肺叶切除术和淋巴结清扫术是最主要的治疗手段之一，而淋巴结清扫术作为重要组成部分，其清扫方式和清扫范围的选择一直备受关注，可能影响术后并发症，尤其慢性咳嗽的发生。本研究旨在探讨淋巴结清扫方式和清扫区域对行肺叶切除术的NSCLC患者术后慢性咳嗽的影响，为优化手术策略、减少术后慢性咳嗽提供临床依据。**方法** 回顾性收集2020年12月至2023年12月中国科学技术大学附属第一医院收治的365例接受肺叶切除术治疗的NSCLC患者临床资料，对其临床特征及术后慢性咳嗽之间的关系进行分析。收集患者术前1天及术后8周2个时间节点的中文版莱斯特咳嗽问卷（Mandarin Chinese version of the Leicester Cough Questionnaire, LCQ-MC）评分数据。根据淋巴结清扫方式分组，探讨淋巴结清扫方式与肺叶切除术后患者慢性咳嗽之间的关系。另外，根据术后是否存在慢性咳嗽分为慢性咳嗽组和非慢性咳嗽组，探讨围手术期资料、淋巴结清扫方式及淋巴结清扫区域是否是其影响因素。**结果** 行肺叶切除术患者中，系统性淋巴结清扫组较淋巴结采样组患者更易发生术后慢性咳嗽（*P*<0.05）。系统性淋巴结清扫组患者术后8周LCQ-MC量表评估显示，其心理、生理、社会及总分均显著低于淋巴结采样组（*P*<0.05）。多因素分析提示：麻醉时间、手术位置、淋巴结清扫方式、是否行上纵隔区淋巴结清扫、上纵隔区淋巴结清扫数目、是否行下纵隔区淋巴结清扫和清扫淋巴结总数目是NSCLC患者术后慢性咳嗽的独立危险因素（*P*<0.05）。**结论** NSCLC患者行肺叶切除术治疗时，选择淋巴结采样较系统性淋巴结清扫可显著降低患者术后慢性咳嗽的发生风险。清扫上纵隔区和下纵隔区淋巴结以及清扫淋巴结的数目可能增加患者术后咳嗽的发生风险，降低患者术后的生活质量。

## Abstract (en)

**Background and objective** Lung cancer has the highest mortality rate among all malignant tumors, and non-small cell lung cancer (NSCLC) accounts for about 80%-85% of all lung cancers. Lobectomy and lymph node dissection are one of the most important treatment methods, and lymph node dissection, as an important part, has attracted much attention. And its mode and scope of dissection may affect postoperative complications, particularly the occurrence of chronic cough. The aim of this study is to investigate the effect of lymph node dissection on postoperative chronic cough in patients with NSCLC undergoing lobectomy, and to provide clinical evidence for optimizing surgical strategy and reducing postoperative chronic cough.**Methods** A retrospective analysis was conducted on the clinical data of 365 NSCLC patients who underwent lobectomy at the First Affiliated Hospital of University of Science and Technology of China from December 2020 to December 2023. The relationship between clinical characteristics and postoperative chronic cough was analyzed. The Chinese version of the Leicester Cough Questionnaire (LCQ-MC) scores were collected from the patients at 2 time points: 1 day before surgery and 8 weeks after surgery. Patients were divided according to lymph node dissection methods, to explore the relationship between lymph node dissection and chronic cough after lobectomy. Additionally, patients were divided into chronic cough and non-chronic cough groups based on the presence of postoperative chronic cough, to investigate whether perioperative data, lymph node dissection methods, and lymph node dissection regions were influencing factors.**Results** Patients undergoing lobectomy were more likely to have chronic cough after surgery in the systematic lymph node dissection group than in the lymph node sampling group (*P*<0.05). LCQ-MC scale evaluation showed that the psychological, physiological, social and total score of the patients in systematic lymph node dissection group were significantly lower than those in lymph node sampling group (*P*<0.05). Multivariate analysis showed that anesthesia time, operation site, lymph node dissection method, whether to perform upper mediastinal lymph node dissection, number of upper mediastinal lymph node dissection, whether to perform lower mediastinal lymph node dissection and total number of lymph node dissection were independent risk factors for postoperative chronic cough in NSCLC patients (*P*<0.05).**Conclusion** When NSCLC patients underwent lobectomy, lymph node sampling was associated with a significantly lower risk of chronic cough than systematic lymph node dissection. Dissecting lymph nodes in the upper and lower mediastinal regions and the number of lymph nodes dissected may increase the risk of postoperative cough and reduce the quality of life of patients after surgery.

肺癌是全球范围内致死率最高的恶性肿瘤^[1,2]^，每年大约有180万例患者死于该疾病^[3]^，其中非小细胞肺癌（non-small cell lung cancer, NSCLC）占所有肺癌的80%-85%^[4]^。在NSCLC的治疗中，肺叶切除术加淋巴结清扫术是最主要的治疗手段之一，其中淋巴结清扫术作为手术的重要组成部分，其清扫方式和清扫范围的选择一直备受关注^[5]^。随着医疗技术的不断进步与革新，患者总体生存期得到显著延长，但肺部手术后咳嗽逐渐成为影响患者术后生活质量的重要因素^[6]^。术后咳嗽指肺部手术后新发或加重的咳嗽，其发生与手术创伤直接相关。慢性咳嗽指咳嗽持续时间超过8周。据统计，肺部手术后出现咳嗽的患者占25%-50%，部分患者如果缺乏早期有效干预会转变为慢性咳嗽，对患者生活质量带来严重影响^[7]^。研究^[8]^显示，肺部手术后咳嗽症状受多种因素共同影响，而淋巴结清扫术作为手术过程中的一个重要环节，被认为可能是引起咳嗽的一个潜在原因。其中，淋巴结清扫方式和清扫区域是否与肺叶切除术后慢性咳嗽有一定关联，目前研究较少且结论存在一定争议，需进一步探讨。因此，本研究旨在探讨不同淋巴结清扫方式和清扫区域与行肺叶切除术的NSCLC患者发生术后慢性咳嗽之间的关系。

## 1 资料与方法

### 1.1 临床资料

本研究回顾性分析2020年12月至2023年12月于中国科学技术大学附属第一医院胸外科接受肺部手术患者的诊疗数据。纳入标准：（1）接受胸腔镜下肺叶切除术；（2）组织病理学证实为NSCLC；（3）R0切除；（4）无新辅助治疗；（5）基于知情基础上的自愿授权。排除标准：（1）术前出现咳嗽症状或合并呼吸道感染性疾病，涵盖感染（如急性呼吸道感染）、慢性炎症（如慢性阻塞性肺疾病、支气管哮喘）及上气道综合征（如鼻后滴流综合征）；（2）手术中转开放手术；（3）采用肺开放性袖式切除术式；（4）术后出现严重不良事件，包括神经损伤、肺栓塞、乳糜性胸腔积液、肺部感染等；（5）未行淋巴结清扫的肺部手术患者；（6）随访依从性差、拒绝接受随访，或病历记录不完整造成数据缺失。本研究经中国科学技术大学附属第一医院医学研究伦理委员会批准（批准号：2023-RE-364）。

经纳入与排除标准筛选后，最终365例患者符合研究条件，其中男性168例，女性197例。基于术中淋巴结清扫方式，全部患者被分为淋巴结采样组（n=128）和系统性淋巴结清扫组（n=237），采集患者基本信息并在指定时间点（术前1天和术后8周）指导患者完成中文版莱斯特咳嗽问卷（Mandarin Chinese version of the Leicester Cough Questionnaire, LCQ-MC）测评。再根据术后咳嗽持续时间是否超过8周，全部患者被分为慢性咳嗽组（n=130）和非慢性咳嗽组（n=235），统计围手术期资料，进行比较。

### 1.2 淋巴结清扫方式

系统性淋巴结清扫参照《中华医学会肺癌临床诊疗指南（2023版）》^[9]^的淋巴结清扫规范，右侧肺癌需清扫2R、4R、7-9及10-14组淋巴结；左侧肺癌需清扫4L、5-9及10-14组淋巴结。

淋巴结采样，作为精准诊断治疗的重要术式，其操作标准为：手术过程中对各区域淋巴结进行系统性探查，当发现可疑转移病灶时，实施整组淋巴结完全摘除，以确保病理评估的完整性；若探查未见明确转移征象的淋巴结，则采取目标区域组织样本送检。该术式通过选择性取样策略，在保证诊断准确性的同时最大程度降低手术创伤。

### 1.3 清扫淋巴结区域

根据国际肺癌研究协会（International Association for the Study of Lung Cancer, IASLC）最新淋巴结分区标准^[10]^，肺癌淋巴结可分7个区域、14组，其转移范围是临床分期的重要依据。根据淋巴结清扫原则，将清扫淋巴结区主要分为以下4组：（1）上纵隔区淋巴结（第2-4组）：包括右上气管旁淋巴结、左上气管旁淋巴结、血管前淋巴结、气管后淋巴结和下段气管旁淋巴结；（2）主动脉区淋巴结（第5-6组）：包括主动脉下淋巴结和主动脉旁淋巴结；（3）下纵隔区淋巴结（第7-9组）：包括隆突下淋巴结、食管旁淋巴结和肺韧带淋巴结；（4）肺门及肺周围区淋巴结（第10-14组）：包括肺门淋巴结、肺叶间淋巴结、肺叶淋巴结、肺段淋巴结及肺段以下淋巴结。

### 1.4 咳嗽评价指标

本研究采用LCQ-MC作为评估工具，系统分析咳嗽对患者生活质量的影响，采用生理、心理和社会3个维度的量表进行全面评估^[11]^，共19个评估项目，其中有8个生理项目、7个心理项目和4个社会项目，每个项目采用7级评分法（正向计分，1-7分，咳嗽程度越轻，分数越高）。各维度得分为对应维度项目均值，总分为3个维度得分之和。本研究纳入的365例患者在2位经过规范化培训的医务人员监督下，分别在术前1天和术后8周2个时间节点进行量表评测。

### 1.5 统计学分析

采用SPSS 26.0统计学软件录入数据，并进行相应统计分析。首先应用Shaprio-Wilk法检验连续变量的正态性，对符合正态分布的变量，采用均数±标准差（Mean±SD）进行统计描述，组间差异比较采用独立样本t检验，不符合正态分布的变量，则用中位数（四分位间距）[M(P_25_, P_75_)]表示，应用Mann-Whitney U法进行组间比较。采用频数（构成比）描述分类变量，组间差异分析使用Pearson卡方检验。慢性咳嗽组和非慢性咳嗽组的比较，首先采用单因素分析，对于单因素分析有统计学意义的变量纳入多因素Logistic回归模型进一步分析。*P*<0.05则提示统计数据差异有统计学意义。

## 2 结果

### 2.1 淋巴结采样组和系统性淋巴结清扫组的一般临床资料比较

淋巴结采样组128例，系统性淋巴结清扫组237例，两组患者在性别、年龄、高血压、糖尿病、术前合并症、第一秒用力呼气容积（forced expiratory volume in one second, FEV_1_）、用力肺活量（forced vital capacity, FVC）、一秒率（FEV_1_/FVC）和最大自主通气量（maximum voluntary ventilation, MVV）方面的差异均无统计学意义（*P*>0.05）（表1）。

**表 1:** 淋巴结采样组和系统性淋巴结清扫组的一般临床资料比较

Variables	Lymph node sampling group (*n*=128)	Systematic lymph node dissection group (*n*=237)	X^2^/t/Z	P
Gender			0.097	0.755
Male	57 (44.53%)	111 (46.84%)		
Female	71 (55.47%)	126 (53.16%)		
Age (yr)			0.217	0.642
<60	71 (55.47%)	124 (52.32%)		
≥60	57 (44.53%)	113 (47.68%)		
Hypertension			0.043	0.835
Yes	35 (27.34%)	61 (25.74%)		
No	93 (72.66%)	176 (74.26%)		
Diabetes mellitus			0.628	0.428
Yes	9 (7.03%)	24 (10.13%)		
No	119 (92.97%)	213 (89.87%)		
Preoperative comorbidity			0.291	0.589
Yes	31 (24.22%)	65 (27.43%)		
No	97 (75.78%)	172 (72.57%)		
FEV_1_ (L)	2.50±0.61	2.42±0.55	1.294	0.196
FVC (L)	3.32 (2.74, 3.90)	3.09 (2.76, 3.61)	-1.702	0.089
FEV_1_/FVC (%)	77.00 (71.00, 81.00)	76.00 (71.00, 79.00)	-0.625	0.532
MVV (L/min)	87.77±19.96	86.66±16.34	0.575	0.566

FEV_1_: forced expiratory volume in one second; FVC: forced vital capacity; MVV: maximum voluntary ventilation.

### 2.2 淋巴结采样组和系统性淋巴结清扫组的咳嗽情况比较（LCQ-MC评分）

术前两组患者LCQ-MC评分在心理、生理、社会及总分方面均无统计学差异（*P*>0.05）。但术后8周系统性淋巴结清扫组在心理、生理、社会及总分方面显著低于淋巴结采样组，差异均有统计学意义（*P*<0.05）。共130例患者术后出现慢性咳嗽，系统性淋巴结清扫组出现慢性咳嗽的患者例数较淋巴结采样组更多，差异具有统计学意义（44.30% vs 19.53%, *χ*^2^=21.175, *P*<0.001）（表2）。

**表 2:** 淋巴结采样组和系统性淋巴结清扫组的LCQ-MC评分及咳嗽情况比较

Variables	Lymph node sampling group (*n*=128)	Systematic lymph node dissection group (*n*=237)	t/X^2^	P
Preoperative				
Physical	6.56±0.20	6.59±0.17	-1.629	0.104
Psychological	6.54±0.15	6.55±0.14	-0.634	0.526
Social	6.55±0.24	6.59±0.22	-1.541	0.124
Total scores	19.35±1.51	19.64±0.34	-1.901	0.060
8 weeks after surgery				
Physical	6.22±0.47	5.89±0.56	5.881	<0.001
Psychological	6.23±0.47	5.89±0.57	6.067	<0.001
Social	6.25±0.96	5.86±0.66	4.087	<0.001
Total scores	18.64±1.36	17.65±1.72	6.097	<0.001
Postoperative chronic cough			21.175	<0.001
Yes	25 (19.53%)	105 (44.30%)		
No	103 (80.47%)	132 (55.70%)		

LCQ-MC: Mandarin Chinese version of the Leicester Cough Questionnaire.

### 2.3 慢性咳嗽组和非慢性咳嗽组的单因素和多因素分析

慢性咳嗽组130例，非慢性咳嗽组235例，两组患者在术后带管时间和肿瘤分期方面差异无统计学意义（*P*>0.05）。在麻醉时间、肿瘤最大径、手术位置、淋巴结清扫方式、上纵隔区淋巴结清扫、主动脉区淋巴结清扫、下纵隔区淋巴结清扫、肺门及肺周围区淋巴结清扫、上纵隔区淋巴结清扫数目、主动脉区淋巴结清扫数目、下纵隔区淋巴结清扫数目、肺门及肺周围区淋巴结清扫数目和淋巴结清扫总数目方面差异有统计学差异（*P*<0.05）（表3）。

**表 3:** 慢性咳嗽组和非慢性咳嗽组的单因素和多因素分析

Item	Univariate analysis				Multivariate analysis				
	Chronic cough group (*n*=130)	Non-chronic cough group (*n*=235)	Z/X^2^	P	B	SE	OR	95%CI	P
Anesthesia time (min)	155 (130, 195)	135 (115, 165)	-4.282	<0.001	0.007	0.003	1.007	1.001-1.013	0.013
Tumor diameter (mm)	20 (13, 25)	15 (10, 20)	3.545	<0.001	0.014	0.013	1.014	0.989-1.041	0.267
Chest tube duration (d)	4 (3, 6)	3 (3, 5)	-1.843	0.066					
Surgical location			39.474	<0.001					0.033
LUL	20 (15.38%)	61 (25.96%)					1.000		
LLL	12 (9.23%)	58 (24.68%)			-0.466	0.445	0.628	0.262-1.503	0.296
RUL	65 (50.00%)	48 (20.43%)			0.703	0.494	2.020	0.767-5.320	0.155
RML	10 (7.69%)	27 (11.49%)			-0.281	0.615	0.755	0.226-2.521	0.648
RLL	23 (17.69%)	41 (17.45%)			-0.109	0.530	0.897	0.318-2.532	0.837
Clinical stage			3.295	0.192					
Stage I	100 (76.92%)	198 (84.26%)							
Stage II	21 (16.15%)	28 (11.91%)							
Stage III	9 (6.92%)	9 (3.83%)							
Lymph node dissection approach			21.175	<0.001					
Lymph node sampling	25 (19.23%)	103 (43.83%)					1.000		
Systematic lymph node dissection	105 (80.77%)	132 (56.17%)			-1.763	0.720	0.171	0.042-0.703	0.014
Superior mediastinal lymph node dissection			33.121	<0.001					
No	16 (12.31%)	99 (42.13%)					1.000		
Yes	114 (87.69%)	136 (57.87%)			1.994	0.689	7.342	1.902-28.345	0.004
Aortic lymph node dissection			9.050	0.003					
No	102 (78.46%)	147 (62.55%)					1.000		
Yes	28 (21.54%)	88 (37.45%)			-0.718	0.558	0.488	0.163-1.455	0.198
Inferior mediastinal lymph node dissection			18.202	<0.001					
No	11 (8.46%)	66 (28.09%)					1.000		
Yes	119 (91.54%)	169 (71.91%)			1.165	0.572	3.206	1.045-9.840	0.042
Hilar and peripheral lymph node dissection			3.922	0.048					
No	3 (2.31%)	17 (7.23%)					1.000		
Yes	127 (97.69%)	218 (92.77%)			0.326	0.712	1.385	0.343-5.594	0.647
Superior mediastinal lymph nodes	5 (2, 8)	2 (0, 4)	7.259	<0.001	0.114	0.043	1.121	1.029-1.220	0.009
Aortic lymph nodes	0 (0, 0)	0 (0, 2)	-2.066	0.049	0.038	0.100	1.039	0.854-1.263	0.704
Inferior mediastinal lymph nodes	4 (2, 7)	3 (0, 5)	3.950	<0.001	-0.006	0.047	0.994	0.906-1.090	0.891
Hilar and peripheral lymph nodes	5 (3, 7)	3 (2,6)	2.847	0.006	0.062	0.041	1.064	0.982-1.153	0.127
Total lymph nodes dissected	17 (12, 21)	10 (4, 16)	6.384	<0.001	0.105	0.073	1.114	1.052-1.219	<0.001

LUL: left upper lung; LLL: left lower lung; RUL: right upper lung; RML: right middle lung; RLL: right lower lung; OR: odds ratio.

将单因素分析有统计学意义的结果纳入多因素分析显示：麻醉时间、手术位置、淋巴结清扫方式、上纵隔区淋巴结清扫、下纵隔区淋巴结清扫、上纵隔区淋巴结清扫数目和清扫淋巴结总数目是肺部手术后慢性咳嗽的独立危险因素（*P*<0.05）（见表3）。

## 3 讨论

咳嗽作为NSCLC患者极为常见的术后并发症，若未能及时干预处理，可能会发展为慢性咳嗽，显著降低患者的生活质量。LCQ-MC问卷在评估肺部疾病患者咳嗽症状的严重程度、发作频率、强度及其对生活质量影响方面展现出良好的可信度。Xie^[12]^和Pan^[13]^等研究表明，LCQ-MC问卷在评价胸腔镜术后NSCLC患者咳嗽症状方面具有有效、简便且可靠的特性。本研究通过比较两组患者的问卷结果发现，术后8周的LCQ-MC评分存在显著差异，系统性淋巴结清扫组的评分较淋巴结采样组更低，且系统性淋巴结清扫组的LCQ-MC评分较术前出现了更为显著的下滑。这一变化趋势证实了淋巴结清扫方式是肺部手术后慢性咳嗽的影响因素，这也与Gu等^[14]^的研究保持一致。

淋巴结清扫术作为NSCLC肺叶切除术中重要的一环，能提高肿瘤术后病理分期的准确性，然而清扫操作可能增加周围组织创伤，导致咳嗽。本研究证明，不同的淋巴结清扫方式是NSCLC患者术后发生慢性咳嗽的独立危险因素。Wu等^[15]^通过比较咳嗽组和非咳嗽组共517例患者的临床资料和治疗结果发现，术中采用系统性淋巴结清扫术的患者比采用淋巴结采样术的患者更易发生术后咳嗽。Chen等^[16]^回顾性分析了196例电视辅助胸腔镜右侧NSCLC根治性切除的临床资料，发现淋巴结采样有效减少了术后慢性咳嗽的发生。淋巴结清扫方式影响患者术后慢性咳嗽的机制主要有以下几种可能：（1）系统性淋巴结清扫范围广，极可能触及喉部、气管、隆突及大的肺支气管的咳嗽感受器和迷走神经分支，此过程潜在地干扰了咳嗽的神经反射通路，从而诱发术后咳嗽反应；（2）系统性淋巴结清扫相比较于淋巴结采样，清扫淋巴结数目较多，手术步骤更多，同时就会伴随着更长的手术与麻醉耗时，以及对气管造成更为显著的额外刺激；（3）系统性淋巴结清扫会导致更大范围的组织创伤，引起持续性的局部炎症，刺激咳嗽感受器；（4）系统性淋巴结清扫中，大量淋巴结清扫可能会破坏淋巴管网，切断肺内淋巴回流途径，导致淋巴液流动阻力增加，造成淋巴液淤积，形成微循环障碍，刺激支气管周围神经和咳嗽反应受体，诱发慢性咳嗽^[17,18]^。Shen-Tu等^[19]^表明，淋巴结采样与系统性淋巴结清扫在肿瘤分期和生存率方面未显示出显著性差异，尤其是在早期NSCLC患者中，淋巴结采样同样能够准确评估淋巴结转移状态。Huang等^[20]^针对早期NSCLC患者的研究表明淋巴结采样与系统性清扫在总生存期、局部复发率及远处转移率方面无统计学差异。因此，对于早期NSCLC患者，淋巴结采样在生存率方面无明显差异的情况下，能够显著降低术后慢性咳嗽的发生率。

本研究通过对慢性咳嗽组与非慢性咳嗽组进行单因素和多因素分析，再次证实淋巴结清扫方式是NSCLC患者术后慢性咳嗽的独立危险因素。在淋巴结清扫区域和数目方面，我们发现上纵隔区淋巴结、下纵隔区淋巴结清扫以及淋巴结清扫数目均被确定为NSCLC患者术后慢性咳嗽的独立危险因素。Huang等^[21]^对肺部手术患者进行气管旁淋巴结清扫并且用自体脂肪填塞，研究发现，肺部手术后咳嗽发生率显著降低。Sawabata等^[22]^对240例肺切除术患者进行了术后咳嗽视觉模拟评分（visual analog scale, VAS）问卷调查并分析，发现纵隔淋巴结清扫是导致术后咳嗽持续存在的因素之一。Wu等^[23]^基于对19项病例对照研究的系统分析，纳入4755例肺切除患者数据，发现纵隔区淋巴结清扫及隆突下淋巴结清扫是肺切除术后咳嗽的独立危险因素。可能的原因为迷走神经C纤维的刺激。迷走神经C纤维是最关键的咳嗽受体，大多数感觉神经纤维包括C纤维，分布在咽喉、气管、隆突、较大的支气管和肺中，而上纵隔区和下纵隔区淋巴结附近就有这些咳嗽受体的分布。淋巴结清扫数目的增加，增加了手术的范围和复杂性，提高了对周围组织尤其是对迷走神经纤维的损伤风险，导致气道敏感增加，术后更易发生慢性咳嗽。为了尽量规避或者减少因过度手术带来的术后慢性咳嗽，选择合理的淋巴结清扫方式，对于需要淋巴结清扫的患者，在不降低手术治疗效果的前提下，尤其在清扫上纵隔区和下纵隔区淋巴结，须尽可能保护迷走神经C纤维和局部组织，以降低术后咳嗽的发生率。

在本研究中，我们还发现手术位置也是术后慢性咳嗽的独立危险因素，这与Xie等^[12]^研究结果一致，右肺叶手术肺切除术后咳嗽的风险更大。Mu等^[24]^认为右上叶切除术可能是肺切除术后持续咳嗽的独立危险因素。原因可能是右肺上叶有更多神经C纤维可能处于无菌性炎症微环境中。

综上所述，行肺叶切除术的NSCLC患者中淋巴结采样较系统性淋巴结清扫导致术后慢性咳嗽的发生率更低，而清扫的淋巴结区域和数目是术后慢性咳嗽的危险因素，尤其上纵隔区和下纵隔区淋巴结清扫会增加术后慢性咳嗽的发生率。因此，针对早期NSCLC患者，术中优先行淋巴结采样有助于降低术后慢性咳嗽的发生率，从而显著提升患者术后的生活质量。但是，本研究存在一定的局限性。首先，这项研究采用小样本、单中心研究设计进行回顾性分析，所选择的病例可能引入了偏倚。因此，当前研究结论需通过多中心、大样本前瞻性随机对照设计验证，以弥补现有证据的局限并增强结论可靠性。另外，本研究未对咳嗽患者的治疗方案进行随访，后续研究应加强这一环节的探索。
